# Bacterial Community and PHB-Accumulating Bacteria Associated with the Wall and Specialized Niches of the Hindgut of the Forest Cockchafer (*Melolontha hippocastani*)

**DOI:** 10.3389/fmicb.2017.00291

**Published:** 2017-02-28

**Authors:** Pol Alonso-Pernas, Erika Arias-Cordero, Alexey Novoselov, Christina Ebert, Jürgen Rybak, Martin Kaltenpoth, Martin Westermann, Ute Neugebauer, Wilhelm Boland

**Affiliations:** ^1^Department of Bioorganic Chemistry, Max Planck Institute for Chemical EcologyJena, Germany; ^2^Center for Sepsis Control and Care, Jena University HospitalJena, Germany; ^3^Leibniz Institute of Photonic TechnologyJena, Germany; ^4^Department of Evolutionary Neuroethology, Max Planck Institute for Chemical EcologyJena, Germany; ^5^Department of Evolutionary Ecology, Institute of Zoology, Johannes Gutenberg University MainzMainz, Germany; ^6^Electron Microscopy Center, Jena University HospitalJena, Germany

**Keywords:** hindgut, *Melolontha hippocastani*, gut bacteria, poly-β-hydroxybutyrate, PHB, *Achromobacter*, Raman microscopy

## Abstract

A characterization of the bacterial community of the hindgut wall of two larval and the adult stages of the forest cockchafer (*Melolontha hippocastani*) was carried out using amplicon sequencing of the 16S rRNA gene fragment. We found that, in second-instar larvae, Caulobacteraceae and Pseudomonadaceae showed the highest relative abundances, while in third-instar larvae, the dominant families were Porphyromonadaceae and Bacteroidales-related. In adults, an increase of the relative abundance of Bacteroidetes, Proteobacteria (γ- and δ- classes) and the family Enterococcaceae (Firmicutes) was observed. This suggests that the composition of the hindgut wall community may depend on the insect’s life stage. Additionally, specialized bacterial niches hitherto very poorly described in the literature were spotted at both sides of the distal part of the hindgut chamber. We named these structures “pockets.” Amplicon sequencing of the 16S rRNA gene fragment revealed that the pockets contained a different bacterial community than the surrounding hindgut wall, dominated by Alcaligenaceae and Micrococcaceae-related families. Poly-β-hydroxybutyrate (PHB) accumulation in the pocket was suggested in isolated *Achromobacter* sp. by Nile Blue staining, and confirmed by gas chromatography–mass spectrometry analysis (GC-MS) on cultured bacterial mass and whole pocket tissue. Raman micro-spectroscopy allowed to visualize the spatial distribution of PHB accumulating bacteria within the pocket tissue. The presence of this polymer might play a role in the colonization of these specialized niches.

## Introduction

Bacteria not only thrive as free-living organisms in the environment, they also engage in complex symbiotic relationships with higher organisms (Wells and Varel, 2011). Insects, in particular, are associated with a large diversity of microorganisms that play important roles for their host’s physiology, ecology, and evolution. The insect gut is colonized by a wide range of bacterial phylotypes that interact with the host and allow it to subsist on nutritionally imbalanced diets. The recycling of nitrogen, the provisioning of essential amino acids and cofactors, and the digesting of recalcitrant polymers in the host’s diet are among the functions for which symbiotic microorganisms play an integral role (Potrikus and Breznak, 1981; Douglas, 2009; Watanabe and Tokuda, 2010), increasing the overall fitness of the insect host.

Typically, the insect gut is divided into three regions, i.e., foregut, midgut and hindgut. The symbiotic bacteria are either attached to the gut wall or colonize the gut as free- living organisms, usually mostly in the mid- and hindgut regions. The structure of these communities differs among insect species, influenced by the host’s diet and taxon (Egert et al., 2003, 2005; Colman et al., 2012; Jones et al., 2013). In the Scarabaeidae family, the hindgut region is of special importance. It is anatomically modified to serve as fermentation chamber. This chamber, in addition to its original function, namely, absorbing water and salts from the gut content, is also devoted to aiding digestion, probably with the help of the fermentative bacteria that colonize it (Egert et al., 2005; Huang et al., 2010; Arias-Cordero et al., 2012; Engel and Moran, 2013). These microbial associates are transmitted either vertically, directly from mother to offspring, or horizontally, that is, being taken anew from the environment by each host generation (Bright and Bulgheresi, 2010). In horizontally transmitted symbiosis, the host usually ingests the symbiont along with unwanted microbes that may compete for the colonization of the gut. The selection of the right symbiont may depend on its phenotypic traits. Kim et al. (2013) showed that poly-β-hydroxybutyrate (PHB) accumulation by the symbiont is crucial for the maintenance of host–microbe relationship.

In this study, we investigate the forest cockchafer (*Melolontha hippocastani*). This scarabaeid constitutes an interesting model due to its particular life cycle, consisting in two well-differentiated stages: the rhizophagous larvae spend up to 4 years underground, while the adults, after pupation, emerge from the soil and shift to a diet based exclusively on foliage. To date, there is a lack of comparative studies on the variation of the gut bacterial community associated with the transition from larva to adult. Only one study addressed this question, a study conducted by Arias-Cordero et al. (2012), focused on the midgut of *M. hippocastani*. Surprisingly, they found a group of bacterial phylotypes that seems to always be stable. This core community is maintained through metamorphosis and is unaffected by the radical change of the host diet from roots to leaves, when the shift occurs from a below-ground (larval) to an above-ground (adult) stage (Arias-Cordero et al., 2012).

In view of this unexpected stability of the gut microbial community, we considered appropiate to characterize the bacterial communities inhabiting the hindgut wall of both below- and above-ground stages of the forest cockchafer, thus complementing the above-mentioned midgut-based study (Arias-Cordero et al., 2012). We put our focus on the hindgut wall itself, and also on particular bacterial niches attached and connected to it, at both sides of the distal part of the larval hindgut. These small structures, called from now on “pockets,” have been hitherto only once described in the literature (Wildbolz, 1954). They consist of several tubular poles connected to the hindgut chamber, which contain bacterial phylotypes that are minor or not detected in the hindgut wall. We detected the presence of PHB within the pockets, and *Achromobacter* sp., one of the major pocket bacterial species, is able to accumulate PHB in pure culture. This suggests that some of the pocket symbionts may be horizontally transmitted, as previous studies found this type of inclusions in symbiotic *Burkholderia* of environmental origin harbored in the midgut crypts of the midgut of *Riptortus pedestris* (Kim et al., 2013). The question of whether PHB plays a role in host nutrition remains unknown.

## Materials and Methods

### Sample Collection and DNA Extraction

**FIGURE 1:**
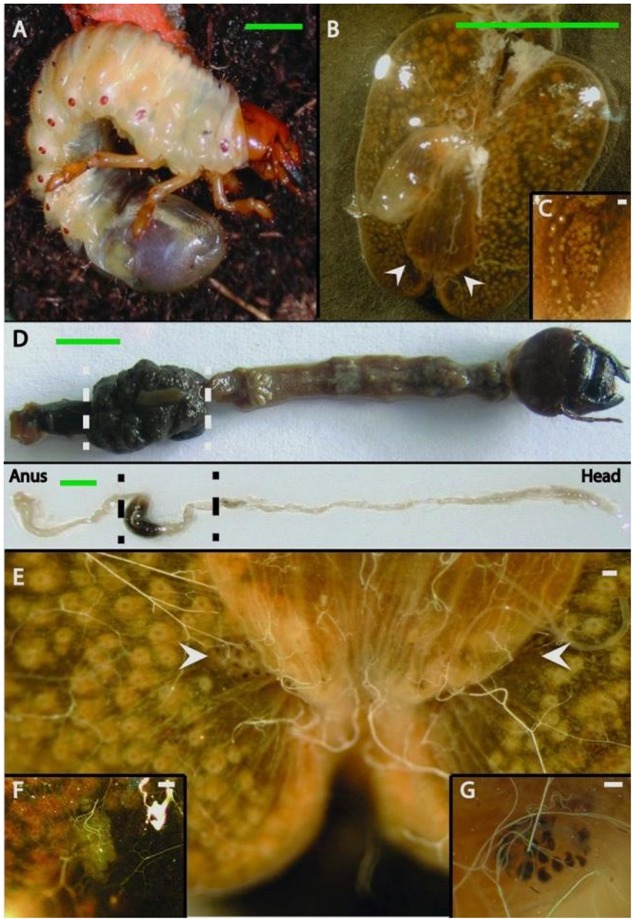
**Gut anatomy of larvae and adults of *Melolontha hippocastani*. (A)** L3 larval instar living in the soil. **(B)** Hindgut fermentation chamber. White arrowheads point to the position of the pockets. **(C)** Close-up of a hindgut lobe. **(D)** Whole gut preparation of an L3 larval instar (top image) and an adult beetle (bottom image). The hindgut section used for microscopy and pyrosequencing is between the dashed lines. **(E)** The fermentation chamber and the pocket position (pointed with arrows). **(F)** Close-up of the *M. melolontha* pocket and **(G)** close-up of the *M. hippocastani* pocket. Scale bars: green 5 mm., white 100 μm.

Second-instar (L2) and third-instar (L3) larvae of *M. hippocastani* and actively flying adults were collected in forests of red oak in Mannheim (49°29′20″N 8°28′9″E), and Graben-Neudorf (49°9′55″N 8°29′21″E), respectively, between December 2010 and May 2014. Beetles were collected at the same sites. The insects were transported alive in boxes with soil or tree leaves. Before dissection, the insects were kept at -20°C for 20 min to kill them, and then rinsed three times alternately with sterile distilled water and 70% ethanol. Dissection was performed on ice in a phosphate-buffered saline (PBS) solution. Hindguts, as shown between dotted lines in **Figure 1D** (top for larva and bottom for adult), were excised, cut open, and carefully washed three times with sterile PBS in order to remove any unattached bacteria. The pockets were separated from the hindgut wall, and as much of the surrounding epithelium was removed as possible. Samples were stored at -20°C before DNA extraction. The day of the extraction, frozen samples were thawed on ice and dried at 45°C for 90 min in a Speedvac (Concentrator 5301, Eppendorf), then crushed in a 1.5 ml tube with a sterile pestle. For 454-pyrosequencing, DNA extractions of the tissue were carried out using the PowerSoil^TM^ DNA Isolation Kit (MO BIO Laboratories Inc., Carlsbad, CA, USA) according to the protocol provided by the manufacturer. Final DNA concentrations were determined using a Nanovue device (GE Healthcare, Little Chalfont, UK). In order to test for the quality of the extracted DNA and confirm the presence of DNA from bacteria, a diagnostic PCR reaction was carried out as described (Arias-Cordero et al., 2012).

### Transmission Electron Microscopy (TEM)

Dissected hindguts and pockets of larvae were fixed in a solution of 2.5% glutaraldehyde and 2% paraformaldehyde in 0.1 M sodium cacodylate buffer (pH 7.2). Immediately afterward, the tissue was transferred to the same solution for overnight fixation. Next day, the fixative was removed, and the tissue was post fixed with 1% osmium tetroxide in cacodylate buffer for 2 h. During the following ascending ethanol series samples were stained with 2% uranyl acetate. The samples were embedded in Araldite CY212 epoxy resin (Agar Scientific Ltd, Stansted, United Kingdom) according to manufacturer’s instruction. Semi-thin sections (1 μm thickness) were stained with Richardson’s methylene blue in order to localize the right position for the examination. Hindgut areas were further trimmed down to 500 μ × 500 μm. Ultra-thin sections of 80 nm thickness were cut using an ultramicrotome Ultracut E (Reichert–Jung, Vienna, Austria) and mounted on Formvar-carbon coated grids (100 meshes, Quantifoil GmbH, Großlöbichau, Germany). Finally, sections were contrasted with lead citrate for 4 min and analyzed in a transmission electron microscope EM900 (Zeiss AG, Oberkochen, Germany).

### Light Microscopy, Richardson Staining, and Autofluorescence Visualization

In all cases the tissue was fixed as described above for transmission electron microscopy (TEM). The tissues employed were larvae hindgut walls and pockets. For the Richardson staining, semi-thin sections of 0.3–0.6 μm (embedded as for TEM) were immersed in a 60°C staining solution for 3–5 min. Afterward, the tissue was washed twice with sterile water. Finally, the sections were placed on a glass slide, dried and mounted for microscopic observation. For the autofluorescence visualization, a excised complete hindgut pocket was placed onto a glass slide and covered with PBS. Visualization was carried out using a LeicaTCS-SP2 confocal microscope using a 10× dry or 40× oil Leica objective (HC PL APO 10×/0.4, Leica, Bensheim, Germany) in both cases. For autofluorescence, laser line employed was 488 nm.

### Bacterial Tag-Encoded FLX Amplicon Pyrosequencing (bTEFAP) and Data Analysis

For pyrosequencing, a sample was composed of the extracted DNA of six insects collected during the same year, pooled together in equal amounts for a single run. A total of four samples were sequenced (L2 pocket, L2 hindgut wall, L3 hindgut wall, and adult hindgut wall). DNA was sent to an external service provider (Research and Testing Laboratories, Lubbock, TX, USA) for bTEFAP with 16S rRNA primers Gray28F (5′-GAGTTTGATCNTGGCTCA-3′) and Gray519R (5′-GTNTTACNGCGGCKGCTG -3′) (Ishak et al., 2011). A sequencing library was generated through one-step PCR with 30 cycles, using a mixture of HotStar and HotStar HiFidelity *Taq* polymerases (Qiagen, Hilden, Germany). Sequencing extended from Gray28F, using a Roche 454 FLX instrument with Titanium reagents and procedures at Research and Testing Laboratory (RTL, Lubbock, TX, USA^1^). Quality control and analysis of 454 reads, including calculation of rarefaction curves and community richness and diversity indexes, was done in QIIME version 1.8.0 (Caporaso et al., 2011). Low-quality ends of the sequences were trimmed with a sliding window size of 50 and an average quality cut-off of 25. Subsequently, all low-quality reads (quality cut-off = 25) and sequences <200 bp were removed, and the remaining reads were denoised using the “denoiser” algorithm as implemented in QIIME (Reeder and Knight, 2010). Denoised high-quality reads were clustered into operational taxonomic units (OTUs) using a multiple OTU picking strategy with cdhit (Li and Godzik, 2006) and uclust (Edgar, 2010), with 97% similarity cut-offs, respectively. For each OTU, the most abundant sequence was chosen as a representative sequence and aligned to the Greengenes core set^2^ using PyNast (Caporaso et al., 2010). RDP classifier was used for taxonomy assignment (Wang et al., 2007). An OTU table was generated describing the occurrence of bacterial phylotypes within the samples.

### qPCR Analysis of Pocket and Hindgut Wall Tissue

For the quantitative real-time PCR (qPCR) analysis, third-instar larvae were used. A sample was composed of the pooled DNA from hindgut wall, or pockets, of three different larval individuals. Three samples from each tissue (hindgut wall and pockets) were considered, and each one was analyzed per triplicate. Specific primers were designed using Geneious 6.0.5^3^ for the five most consistently found bacterial taxa in the pocket (*Achromobacter*, *Citrobacter*, *Bosea*, *Brevundimonas*, and *Pseudomonas*), based on the alignment of the representative set of sequence data for all OTUs available from the 454-pyrosequencing. PCR conditions for each primer pair were optimized using gradient PCRs (Salem et al., 2013). Their specificity was verified *in silico* against the SILVA ribosomal RNA database^4^ and *in vitro* by sequencing. Briefly, PCR products from pocket DNA were analyzed on 1% agarose gels (150 V, 30 min). The products were purified from the gel with Invisorb Fragment CleanUp kit (Stratec Molecular, Berlin, Germany) and cloned in pCR 2.1 vector using the Original TA Cloning kit (Invitrogen, Carlsbad, CA, USA). Ninety clones with positive inserts were selected according to the manufacturer’s protocol and sequenced on a 3730 XL DNA Analyzer (Applied Biosystems, Foster City, CA, USA) with BD 3.1 chemistry. If the sequence matched the expected OTU, the primer pair was assumed to specifically amplify the target OTU within the gut and pocket. The sequences of the primers are listed in Supplementary Table S2. Quantitative PCRs for individual bacterial taxa were performed on a CFX96 Real Time System (Bio-Rad, Munich, Germany), in final reaction volumes of 10 μL containing 1 μL of template DNA (usually a 1:10 dilution of the original DNA extract), 0.6 μL of each primer (10 pM) and 5 μL of SYBR Green Mix (Rotor-Gene SYBR Green kit, Qiagen, Hilden, Germany). Standard curves were established using 10^-6^–10^-2^ ng of specific PCR product as templates for the qPCR. A NanoDrop ND-1000 spectrophotometer (Peqlab Biotechnology Limited, Darmstadt, Germany) was used to measure template DNA concentration for the standard curve. Five different replicates of the standard concentrations for each bacterial taxon were used to calculate a correction factor and determine equitation parameters. PCR conditions were as follows: 95°C for 3 min, followed by 40 cycles of denaturation at 95°C for 10 s, annealing for 30 s and elongation at 72°C for 10 s. Then, a melting curve analysis was performed to ensure that amplicons were the same across samples for each primer assay, by increasing the temperature from 65 to 95°C within 5 min. The annealing temperature was specific for each primer pair: for *Achromobacter* and *Citrobacter*, 60°C; for *Bosea*, 63°C; for *Brevundimonas*, 55°C; for *Pseudomonas*, 68°C. Based on the standard curves, the 16S copy number could be calculated for each individual sample from the qPCR threshold values (Ct) by the absolute quantification (Lee et al., 2006, 2008), taking the dilution factor and the absolute volume of DNA extract into account. The quantitative differences in the microbial community abundances of the pocket were tested using SPSS 17.0 (Tukey HSD test, confidence interval of 0.05).

### Isolation and Identification of Pocket Bacteria

Four second-instar pockets from different larvae were dissected as mentioned above and incubated together in a 0.8% NaOCl aqueous solution for 3 min on ice for surface sterilization. Then, the tissue was transferred in Ringer+ppi buffer (Cazemier et al., 1997) and sonicated using a Sonorex Super RK 102h sonicator (Bandelin, Germany) for 7 min at RT. After sonication, the tubes were incubated 15 min on ice and gently tapped from time to time. Ten-fold dilutions of the supernatant were plated on LB agar (Carl Roth, Germany) and ATCC agar in order to enrich for *Achromobacter* sp. The ATCC agar contained (per liter): 7.32 g K_2_HPO_4_, 4.6 g ammonium tartrate, 1.09 g KH_2_PO_4_, 0.04 g MgSO_4_ 7H_2_O, 0.04 g FeSO_4_ 7H_2_O, 0.014 g CaCl_2_ 2H_2_O, and 35 g agar. Plates were incubated at 30°C for 48 h. Morphologically different colonies were subcultured three times before identification. Colony PCR targeting the small ribosomal subunit gene was performed on a GeneAmp 9700 Thermocycler (Applied BioSystems) using the general bacterial primers 27f and 1492r (Arias-Cordero et al., 2012). The 50 μL reaction mixture contained 1x buffer, 1.5 mM MgCl_2_, 10 mM of the four deoxynucleotide triphosphates (dNTPs), 2.5 U Taq DNA polymerase (Invitrogen) and 0.5 mM of each primer. The PCR program was as follows: initial denaturation at 94°C for 3 min followed by 32 cycles of denaturation at 94°C for 45 s, annealing at 55°C for 30 s and elongation at 72°C for 1 min, and a final elongation step at 72°C for 10 min. Amplicon size was confirmed in a 1% agarose gel; then the PCR product was purified using the Invisorb Fragment CleanUp kit (STRATEC Molecular GmbH, Berlin, Germany). Sequencing was performed at Macrogen Europe (Amsterdam, The Netherlands), and the taxonomy of resulting sequences was assigned using Basic Local Alignment Search Tool (BLAST) (Tatusova and Madden, 1999).

### Metabolic Testing of Bacterial Isolates

Nile Blue agar was prepared as described (Luellen and Schroth, 1994). A representative of each bacterial isolate was plated and incubated for 48 to 72 h at 30°C. The plates were then viewed under UV light to detect putative PHB production based on the fluorescence of the colonies. Nitrate reduction test was purchased from Sigma and conducted following the instructions provided by the manufacturer. A representative of each bacterial isolate was inoculated at high density, and tubes were sealed with liquid paraffin to create oxygen-poor conditions and incubated at 30°C up to 5 days.

### Gas Chromatography – Mass Spectrometry

Twenty-five third-instar larvae were dissected as described, and their 50 pockets were analyzed as one single sample. *Achromobacter* sp. isolated from the pocket was cultured for 3 days in PHB inducing broth at 30°C for 72 h. The composition of PHB inducing broth is the same as Nile Blue agar (Luellen and Schroth, 1994) without Nile Blue or agar. The bacterial mass was recovered by centrifugation and washed twice with sterile distilled water prior to drying [45°C for 90 min in a Speedvac (Concentrator 5301, Eppendorf)]. 5 mg (dry weight) of bacterial mass was used for the analysis. Poly[(*R*)-3-hydroxybutyric acid] standard was obtained from Sigma (Germany), and 1 mg was used for the analysis. Derivatization was performed as described (Riis and Mai, 1988), using methanol instead of propanol for the esterification. GC analysis was performed in a ThermoQuest, Finnigan Trace GC-MS 2000 series (Egelsbach, Germany), equipped with a fused-silica capillary Phenomenex ZB-5 column (15 m × 0.25 mm, film thickness 0.25 μm) with a split ratio of 10:1. Helium was used as carrier gas at a flow rate of 1.5 ml/min. The oven temperature was programmed as follows: the initial temperature of 60°C was held for 3 min, then increased to 230°C at 30°C/min and held for 2 min. The inlet temperature was 250°C and the injection volume 1 μL. Mass spectra were measured in electron impact (EI) at 70 eV under full scan mode (m/z 35–575). Acquired data were further processed using the software Xcalibur (Thermo Scientific). 3-hydroxybutyric acid methyl esters were identified by comparison of the mass spectrum and retention time with poly[(*R*)-3-hydroxybutyric acid] standard.

### Raman Micro-Spectroscopy

One pocket was used for each Raman measurement. CaF_2_ slides suitable for Raman spectroscopy were poly-L–lysine coated by being soaked overnight in 0.1% poly-L–lysine solution (Sigma) at 4°C prior to measurement. The pocket tissue was fixed overnight with 4% paraformaldehyde solution in 0.9% NaCl at 4°C. After fixation, the paraformaldehyde was removed and the tissue was washed three times for 10 min with 0.9% NaCl solution under mild agitation. Then the pocket tissue was embedded in a mounting medium for cryotomy, OCT compound (VWR Chemicals, Radnor, PA, USA) and sliced in 12-μm thick sections using a Microm HM 560 cryomicrotome (Thermo Scientific, Waltham, MA, USA). The tissue slices were put onto the poly-L–lysine coated CaF_2_ slide, washed carefully with 0.9% NaCl to remove the remains of the mounting medium and viewed under a bright-field microscope to check for the characteristic round-shaped cross-sections of the pocket poles. The Raman spectra were acquired with a confocal Raman microscope alpha 300R (WITec, Ulm, Germany) using a 532 nm Nd:YAG solid laser with a power of 15 mW for excitation. The samples were measured in 0.9% NaCl using a 60× water immersion objective with NA 1.0 (Nikon, Tokyo, Japan). Collection of backscattered photons occurred through a back-illuminated CCD camera (DV401-BV-352, Andor, Belfast, UK). For spectral grating, 600 lines/mm were used for 532 nm. A multimode fiber of 25 μm diameter served as pinhole for confocal imaging. The Raman spectra were recorded by using 1 s integration time. Characteristic spectra and compartments in the pocket poles were detected by analyzing the Raman scans with the N-FINDR unmixing algorithm (Winter, 1999; Hedegaard et al., 2011) using Matlab software (MathWorks). The PHB was detected by identifying specific peaks through comparison with measured reference spectrum of pure PHB compound.

### Nucleotide Sequence Accession Numbers

The 16S RNA gene sequences obtained by colony PCR have been deposited at the NCBI GenBank under accession numbers from KY178280 to KY178284 (**Table 1**). Pyrosequencing data from L2 hindgut wall, adult hindgut wall, L2 pocket and L3 hindgut wall have been deposited under accession numbers SRR5059348, SRR5059349, SRR5059340, and SRR5059351, respectively.

**Table 1:** Bacterial isolates from *Melolontha hippocastani’s* pockets with their metabolic capabilities.

Closest taxonomic affiliation	Identity percentage	Nile blue staining	Denitrification	Accession number
*Citrobacter murliniae*	99	Negative	Nitrate to nitrite	KY178281
*Achromobacter marplatensis*	99	Positive	Nitrate to nitrogen	KY178280
*Ochrobactrum thiophenivorans*	100	Negative	Negative	KY178282
*Phyllobacterium myrsinacearum*	99	Positive	NT	KY178283
*Stenotrophomonas maltophilia*	99	Positive	NT	KY178284


*NT, not tested.*

## Results

### Localization and Morphology of the Pockets

**FIGURE 2:**
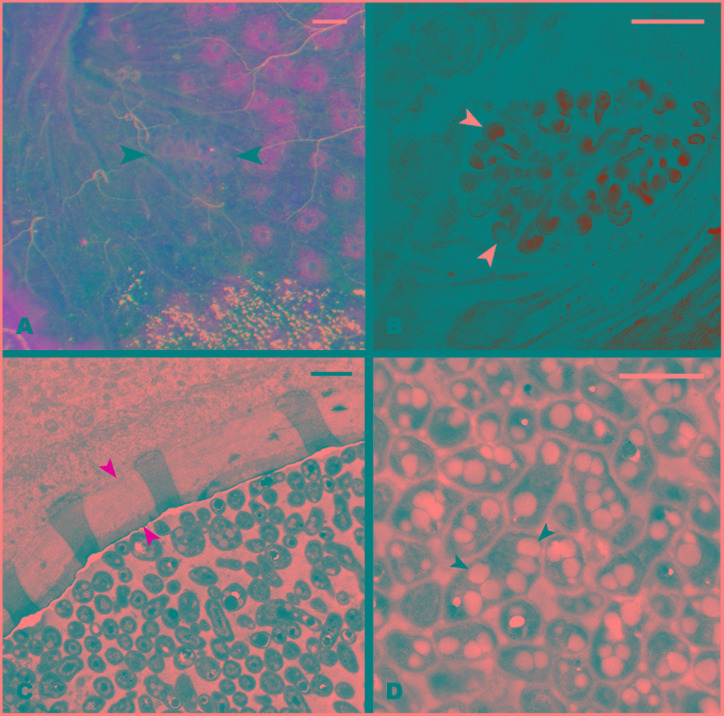
**Structure of the hindgut pocket. (A)** Pocket attached to the hindgut external surface (black arrowheads). **(B)** Autofluorescence image of the pocket tissue using a 488 nm laser in a confocal microscope. White arrowheads point to pocket poles. **(C)** Transmission Electron microscopy (TEM) image of a cross-section of a pocket pole. The yellow arrowheads point to the acellular layer, possibly mucous-like, enveloping every pole. **(D)** TEM image of the dense bacterial population in the center of the pocket poles. Black arrowheads point to the PHB granules observed in the bacterial cytoplasm. Scale bars: **(A,B)** 100 μm, **(C,D)** 1 μm.

During the dissection of larval individuals (**Figure 1A**), two small structures [“pockets,” colored either white or black (**Figures 1F,G**)] attached outside the terminal point of the hindgut chamber (**Figures 1B,C,D,E**, **2A**) were spotted. The pockets have a diameter of around 500 μm, and showed high autofluorescence when illuminated with a 488 nm laser (**Figure 2B**). They are covered by a fine layer of muscle tissue (Supplementary Figure S1). Their anatomy is composed by poles connected to the hindgut lumen (Supplementary Figure S2). Further anatomical investigation by TEM revealed that each pole was surrounded by a thick acellular tissue layer (possibly mucous-like, **Figure 2C**). Additionally, it was observed that each pole was lined with large numbers of bacterial cells (**Figure 2C**). These cells showed a high number of cytoplasmatic inclusions (**Figure 2D**).

### Pyrosequencing of the Bacterial Community from the Hindgut Wall of Adult Insects and Larvae, and Pockets

**FIGURE 3:**
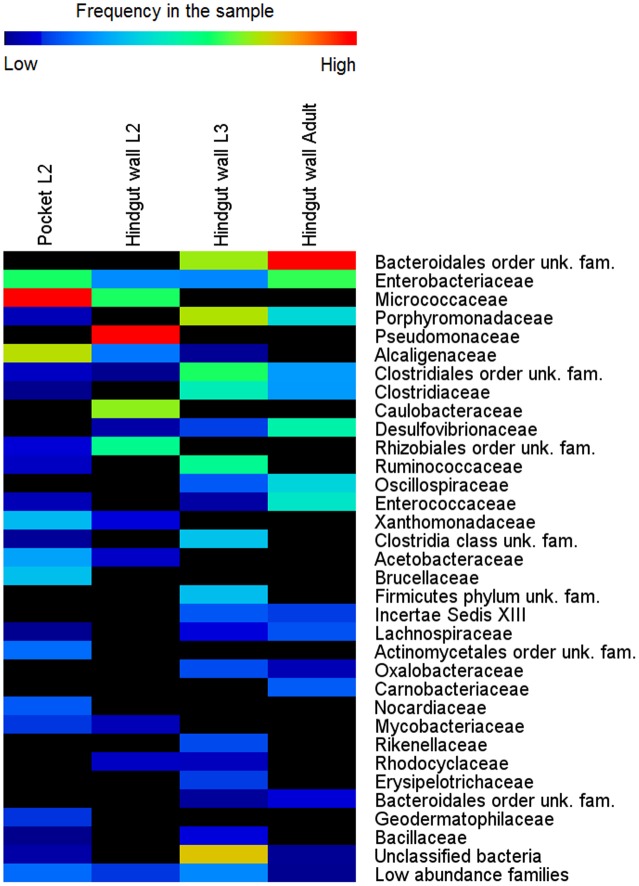
**Bacterial community composition in different life stages of *M. hippocastani*.** Relative abundance of bacterial taxa, in percentage of total sample sequences, from 454 pyrosequencing data (110,772 sequences in total) is displayed as a heat map based on the log-transformed values. Warm colors indicate higher and cold colors lower abundances. Families with total relative abundance lower than 0.4% were considered “low abundance families” and listed in Supplementary Table S3. unk., unknown; fam., family.

To establish the dynamics of the hindgut wall community across different host’s life stages, the bacterial communities of the hindgut wall of L2 and L3 larvae and adults were compared. DNA from six different insects of each life stage was used, pooled together in a single pyrosequencing run. In the final output, 110,772 high quality reads were obtained (Supplementary Table S1). It was found that, in the L2 hindgut wall, the main bacterial phyla were Pseudomonadaceae, Caulobacteraceae and Micrococcaceae, while in L3 hindgut wall, those were Bacteroidetes phylum and Clostridia, with a large proportion of unknown bacteria. In the adults, an increase of the relative abundance of the Bacteroidales order, Proteobacteria (γ- and δ- classes) and the family Enterococcaceae (Firmicutes) was observed (**Figure 3**). Estimation of alpha-diversity in these samples was done using rarefaction methods, and richness and diversity indexes were also calculated (Supplementary Figure S3 and Supplementary Table S1).

Amplicon sequencing revealed considerable differences in microbial communities between the L3 and L2 hindgut walls. In L2, approximately 47% of the sequences obtained belong to the family Pseudomonadaceae and 30% to the family Caulobacteraceae, taxa that were not detected in the L3 hindgut wall; the L3 hindgut wall, in turn, had families at high abundances which were not or only at low abundances detected in L2 (e.g., Porphyromonadaceae, Bacteroidales, and Ruminococcaceae) (**Figure 3**). This may reflect the changes that the bacterial community undergoes throughout the different stages of the insect’s life, suggesting that the hindgut wall is a dynamic environment.

A pooled sample of DNA extracted from 12 excised pockets (from 6 L2 larvae) was also sequenced, in order to compare their bacterial communities with the surrounding hindgut wall. It was found that the main bacterial phyla of the pocket tissue were Actinobacteria and Proteobacteria (α- and β- classes). Within the β-Proteobacteria, *Achromobacter* sp., which accounted for 85% of sequences from the family Alcaligenaceae, was the genus with the overall highest relative abundance in the pockets. The classification at genus level of the family Micrococcaceae was not achieved. These two families were present in low abundance in the L2 and L3 hindgut wall, as well as in the hindgut wall of adult beetles (**Figure 3**).

### Estimation of Absolute Abundances of Main Bacterial Genera in the Pockets and the Hindgut Wall

**FIGURE 4:**
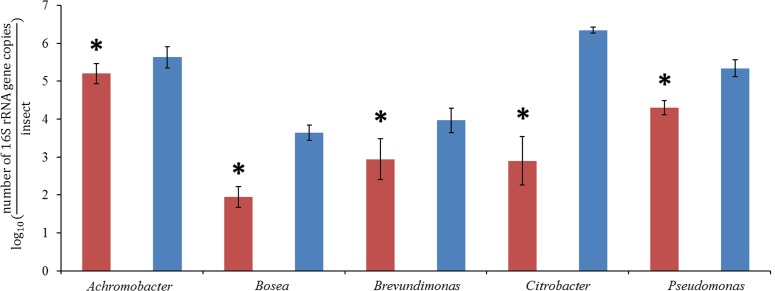
**quantitative real-time PCR (qPCR) of pocket samples (red) and hindgut wall samples (blue), obtained with genus-specific primers for *Achromobacter*, *Bosea*, *Brevundimonas*, *Citrobacter*, and *Pseudomonas* (ANOVA, Tukey HSD test, ^∗^*p* < 0,05, ±1 SD, *n* = 10)**.

In order to compare the absolute abundances of key genera inhabiting the pocket and the hindgut wall of L3 larvae, namely *Achromobacter* (family Alcaligenaceae), *Bosea* (family Bradyrhizobiaceae), *Brevundimonas* (family Caulobacteraceae), *Citrobacter* (family Enterobacteriaceae) and *Pseudomonas* (family Pseudomonadaceae), qPCR with genus-specific primers was performed. In the pocket, *Achromobacter* was the most dominant of the genera, with an abundance about 10 times greater than that of *Pseudomonas* (**Figure 4**). *Citrobacter, Brevundimonas*, and *Bosea* showed lower abundances, with that of *Bosea* being three orders of magnitude lower than that of *Achromobacter*. The abundances of all four lower-abundant genera in the pockets differed significantly from that of *Achromobacter* (ANOVA, Tukey HSD test, *p* < 0.05). This is in line with the outcome of the 454-pyrosequencing, in which *Achromobacter* sp. (85% of family Alcaligenaceae sequences) was the most dominant of the identified genera in the pocket (**Figure 3**). However, since it was not possible to classify the family Micrococcaceae at the genus level, it must be taken into account that *Achromobacter* sp. may be overcome by a Micrococcaceae-related genus.

In the hindgut wall, the abundances of *Pseudomonas, Brevundimonas*, and *Bosea* spp. (*Pseudomonas* > *Brevundimonas* > *Bosea*) were in good agreement with their respective family abundances showed by the 454 pyrosequencing approach. The occurrences of *Citrobacter* and *Achromobacter* spp., respectively, the first and second most ubiquitous genera according to the qPCR outcome, matched their respective abundances in the L3 hindgut wall pyrosequencing (families Enterobacteriaceae and Alcaligenaceae, respectively), but were significantly higher than their abundances in L2 hindgut wall pyrosequencing (**Figure 3**). This outcome fits with the abovementioned idea that the relative abundances of the gut bacterial community members are dynamic depending on the larval instar.

### PHB Detection in Pocket Isolates and Pocket Tissue by Nile Blue Staining and GC-MS

Considering the relatively close phylogenetic relationship between the major genus in *M. hippocastani* pockets, *Achromobacter* sp., and the PHB-accumulating bacterium that colonizes the *R. pedestris* midguts crypts, *Burkholderia* sp. (Kim et al., 2013), we speculated that PHB accumulation could also take place in the pocket symbionts. To test this hypothesis, pocket symbionts were isolated in selective media. The bacterial species that were retrieved are listed in **Table 1**. PHB accumulation was suggested in *Achromobacter marplatensis*, *Stenotrophomonas maltophilia*, and *Phyllobacterium myrsinacearum* by its positive fluorescence under UV light when cultured in Nile Blue agar (**Table 1**) (Ostle and Holt, 1982).

**FIGURE 5:**
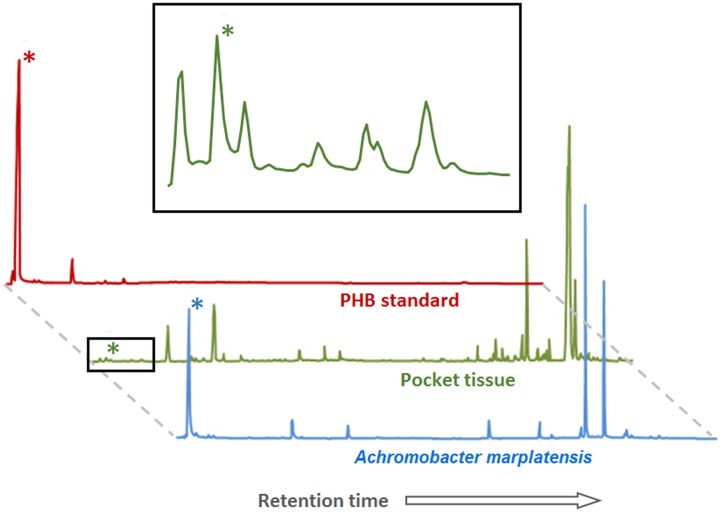
**Poly-β-hydroxybutyrate (PHB) detection by GC-MS in pocket tissue and *Achromobacter marplatensis* isolated from the pocket**. The peak height reflects the relative abundance of the substance. Peaks marked with (^∗^) correspond to the methyl ester of the 3-hydroxybutyric acid monomer of PHB. The area marked with a black square is enlarged above. The other peaks correspond to a variety of fatty acids of different chain lengths, common in both eukaryotic and prokaryotic cells, and to artifacts created by the method (peaks in the PHB standard chromatogram).

Gas chromatography coupled with mass spectrometry (GC-MS) of pocket tissue as well as isolated *A. marplatensis* was conducted in order to confirm PHB presence. For the analysis, pockets and bacterial mass were derivatized through *trans*-esterification with methanol in the presence of acid (see Materials and Methods) prior to injection into the gas chromatograph. The resulting chromatograms (**Figure 5**) showed a peak corresponding to 3-hydroxybutyric acid methyl ester, the derivatized 3-hydroxybutyric acid monomeric unit of PHB, with a retention time of 2.21 min (±0.01 min). Its identification was carried out by comparing the obtained mass spectrum and the retention time with the commercially available reference compound.

### Raman Micro-Spectroscopy of the Pocket Tissue

**FIGURE 6:**
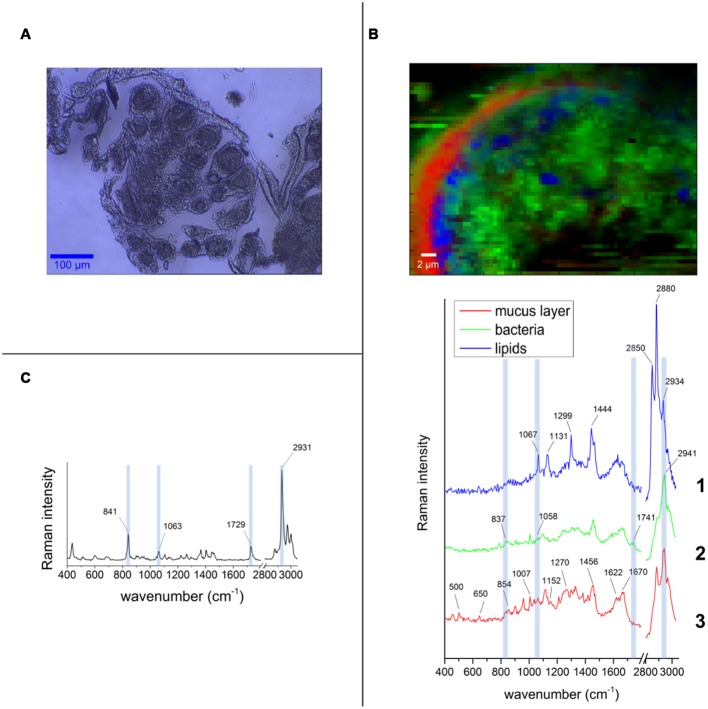
**False-color Raman images and spectra obtained from the pocket tissue with 532 nm excitation wavelength. (A)** Bright field image of one of the tissue cuts used for the measurements. The round shape of the pocket poles is clearly visible. **(B)** Pocket pole with PHB-containing bacteria (green area in false-color image, spectrum 2). Spectrum 1 corresponds to fatty acids (blue area in false-color image) and spectrum 3 corresponds to the acellular mucus-like layer that surrounds the inner part of the pole epithelium (red area in false-color image). **(C)** Spectrum of the PHB pure substance is used as standard compound. The bands that are distinguishable in the spectrum from the pocket pole are highlighted in light blue.

In order to determine the spatial distribution of PHB-accumulating bacteria within the pocket pole, Raman micro-spectroscopy was performed. The Raman spectroscopic scans and spectra obtained are shown in **Figure 6**. Spectral unmixing using the N-FINDR algorithm revealed false-color images that showed different constituents by identifying different Raman spectral signatures. In the pocket poles containing PHB-accumulating bacteria, they were distributed uniformly throughout the inner area of the pole as dots of approximately 1 μm diameter (spectrum 2 of **Figure 6B**, green area in false-color image). Within a typical bacterial Raman spectrum (Ciobotǎ et al., 2010; Majed and Gu, 2010), the presence of PHB granules was indicated by the bands at 837 and 1058 cm^-1^ (C-C stretching), and especially by the highly significant band at 1741 cm^-1^ (C=O stretching; compare PHB reference spectrum in **Figure 6C** with spectrum 2 in **Figure 6B**). The spectrum showing mainly C-C stretching (1067, 1131 cm^-1^), CH_2_ twisting (1299 cm^-1^), and CH_2_ bending (1444 cm^-1^) vibrations (spectrum 1 in **Figure 6B**, blue area in false-color image), were likely derived from fatty acids, probably of a saturated nature as the bands that provide evidence of unsaturation were missing (1260, 1650, and 3023 cm^-1^), whereas the bands that support saturation were strong (1299, 1444, CH stretch region at 2800 – 3000 cm^-1^) (Wu et al., 2011). Finally, the spectrum of the mucus-like layer (**Figure 2C**) surrounding the inner part of the pole (spectrum 3 of **Figure 6B**, red area in false-color image) revealed a complex composition, consisting mainly of proteins with disulphide bridges (S-S stretch, band 500 and 505 cm^-1^, respectively), high tyrosine (Tyr) content with bands at 650 cm^-1^ (C-C twist Tyr), 854 and 859 cm^-1^ (ring vibration Tyr), 1270 cm^-1^ (protein amide III), 1456 cm^-1^ (CH_2_ deformation), 1622 and 1626 cm^-1^, respectively (C=C stretching Tyr and Trp), 1670 cm^-1^ (protein amide I or C=C stretching) (Tuma, 2005), and lipids (band 1270 cm^-1^ CH bend), and 1456 cm^-1^ (CH_2_ deformation). For more detailed band assignment information, see Supplementary Table S4.

## Discussion

### Bacterial Communities of the Hindgut Wall and the Pockets

Four hundred and fifty-four-pyrosequencing revealed that, in L2 larvae, the bacterial community of the hindgut wall was dominated by the families Pseudomonadaceae and Caulobacteraceae. These families, however, were overgrown in L3 by representatives of the family Porphyromonadaceae and the orders Bacteroidales and Clostridiales. Since these taxa are anaerobic, their proliferation in late larval instars may reflect a thickening of the bacterial layer attached to the hindgut wall, allowing the symbionts to reach more anaerobic areas toward the hindgut lumen, or a pronounced decrease in oxygen concentration due to high bacterial density. Similar shifts in bacterial abundances depending on the maturity of the larvae have been previously reported by Zheng et al. (2012) in *Holotrichia parallela* larvae. The hindgut wall of these larvae is populated by a reduced amount of coccoid cells in the L1 stage, although in the L3 stage, the density of bacteria is largely increased, with bacteroid cells dominating (Zheng et al., 2012).

In the adults, the relative abundances of Bacteroidetes, Proteobacteria (γ- and δ- classes) and the family Enterococcaceae (Firmicutes) were increased. Nevertheless, the overall composition of the adult hindgut wall community remained fairly constant compared to L3. This is in line with previous observations on *M. hippocastani* (Arias-Cordero et al., 2012). It was noted that the similarity between larval and adult bacterial communities becomes more evident in the later larval instars, suggesting that L3 larvae possess a community that is more closely related to that of the adults than to the L2 larvae. In addition, they noticed that the abundance of Enterobacteriaceae in the midgut increased continuously throughout the L2, L3, and adult stages (L2 < L3 < adult). In line with these findings is the presently observed increase of the genus *Citrobacter* from L2 to L3 (**Figures 3**, **4**). Such increase in abundance of *Citrobacter* representatives toward latter larval instars may be related to the increasing amount of ingested food as the larvae grow, as previously isolated *Citrobacter* sp. from the gut of *M. hippocastani* showed the ability to degrade xylan and starch in pure culture (Arias-Cordero et al., 2012). Furthermore, in adults, the high abundance of Enterococcaceae and Enterobacteriaceae representatives might be related to the shift to leaf-based diet, as these families showed resistance to tannins, an ubiquitous plant defense compound (Smith and Mackie, 2004; Singh et al., 2011).

The abundances of the bacterial genera in the L3 hindgut wall showed by qPCR (**Figure 4**) are in good agreement with the pyrosequencing result, being *Citrobacter* sp. dominant over *Achromobacter* sp., just as the Enterobacteriaceae family is more abundant than Alcaligenaceae in **Figure 3**. Contrary, *Achromobacter* sp. dominates in the pocket. This is also in line with the 454-pyrosequencing, where the sequences obtained clustered mainly within Actinobacteria and α- and β-Proteobacteria, taxa that showed very low abundances in the hindgut wall. This result highlights the singularity of the pocket bacterial community and suggest that they function as specialized symbiotic niches, analogously to previously described structures in other insects (Kikuchi et al., 2005; Grünwald et al., 2010).

### Significance of the PHB Inclusions

Transmission electron microscopy unveiled a number of white cytoplasmatic inclusions in the pocket bacteria [potential poly-3-hydroxybutyrate (PHB)]. By GC-MS analyses, it was possible to confirm the presence of poly-3-hydroxybutyrate. Raman micro-spectroscopy revealed that PHB-accumulating bacteria are widely distributed throughout the lumen of the pocket pole. PHB is commonly accumulated by Eubacteria and Archaea and serves as a carbon reserve, stored in the form of water insoluble droplets in the cytoplasm (Rehm, 2003). Its presence is probably linked to the white cytoplasmatic inclusions observed in TEM. Likewise, PHB inclusions also are present in the endosymbiont *Burkholderia* sp. colonizing the midgut crypts of the bean bug *R. pedestris.* Each generation of this insect orally acquire the *Burkholderia* bacterium *de novo* from the environment, and the accumulation of PHB by the symbiont is crucial to ensure proper colonization of the crypts and correct development of the insect host (Kim et al., 2013). The colonization success by the PHB-accumulating symbiont could be related to its enhanced ability to cope with stress, as previous studies linked PHB accumulation to an increase of bacterial colonization efficiency and to tolerance to a variety of stresses such heat, reactive oxygen species, osmotic imbalance and nutritional depletion, among others (Kadouri et al., 2003; Kim et al., 2013). The high lipidic content within the pocket pole revealed by Raman micro-spectroscopy (**Figure 6**), suggests that the pockets are a nutritionally imbalanced habitat with a high C:N ratio that may favor the colonization by bacteria with the ability of accumulate PHB (Rehm, 2003). Also, oxygen limitation might contribute on selecting PHB-accumulating bacterial species over non-accumulating ones (Trainer and Charles, 2006). Symbiont sorting mechanisms in order to discard potentially pathogenic bacteria from the soil have been reported in the bean bug *R. pedestris* (Kim et al., 2013; Ohbayashi et al., 2015). However, in *M. hippocastani*, this putative discriminative process would not be as specific as in *R. pedestris*, since more than one bacterial phylotype are established in the pockets.

The presence of PHB is uncommon in vertically transmitted bacterial symbionts. Its accumulation is displayed mainly by free-living microorganisms, or by symbionts of environmental origin (Kim et al., 2013). This suggests that the PHB-accumulating pocket symbionts (*A. marplatensis* and possibly *S. maltophilia* and *P. myrsinacearum*; see **Table 1**) might be acquired from the environment. These genera, along with *Ochrobactrum thiophenivorans* (which is not likely to accumulate PHB; see **Table 1**), have been previously detected in the rhizosphere (Bertrand et al., 2000; Kämpfer et al., 2008; Ryan et al., 2009). Moreover, the BLAST alignments of the pocket isolates belonging to these taxa matched those of bacteria previously isolated from roots and soil (data not shown). Considering that, an environmental origin for these pocket symbionts is more plausible than a vertical transmission from mother to offspring. This latter possibility, nevertheless, cannot be totally discarded (Engel and Moran, 2013).

### Physiological Role of the Pockets

The pockets in *M. hippocastani* have been only once described in literature (Wildbolz, 1954). Nonetheless, symbioses between insect and bacteria is a common and disparate phenomenon in nature (Douglas, 2009; Hansen and Moran, 2014) and analogous structures harboring symbiotic microorganisms have been found in other insects. Bugs belonging to the family Alydidae are associated with ectosymbiotic bacteria of the genus *Burkholderia.* It this case, the bacterium colonizes the crypts located in the distal section of the midgut (Kikuchi et al., 2005). Similarly, stinkbugs of the families Pentatomidae and Cydnidae harbor Gammaproteobacteria related bacteria in crypts located in the same region of the midgut (Prado and Almeida, 2009; Hosokawa et al., 2012). Other structures containing endosymbiotic bacteria and yeasts have been characterized in the proximal midgut of cerambycid beetles (Grünwald et al., 2010). The role of these symbionts within the insect gut and their involvement in host’s nutrition, however, remains largely unknown.

In *M. hippocastani*, the pockets might be sites for denitrification processes. *A. marplatensis* isolated from these small structures showed full denitrifying capabilities in a commercial nitrate reduction assay (**Table 1**). Moreover, the abundance of lipids within the pocket pole unveiled by Raman micro-spectroscopy (**Figure 6**) makes possible that these compounds are used by the pocket symbionts as electron donors for respiratory processes using nitrate as an electron acceptor (NO_3_^-^). Denitrification has already been reported in other rhizophagous white grubs (Majeed and Miambi, 2014). The presence of pockets could be also related to the rhizophagous diet of the larvae, as they were spotted in the rhizophagous larvae of *M. melolontha* as well (**Figure 1F**), but no similar structure was found in *Pachnoda marginata* (Supplementary Figure S4), whose grub-like larvae thrive not on roots but on humic acids. Host’s diet and taxonomy have been pointed as key determinants of the composition of the gut symbiotic community by previous studies (Egert et al., 2003, 2005; Colman et al., 2012; Jones et al., 2013). Either way, it is possible that the pocket symbionts produce some kind of beneficial compound for the insect host. This hypothesis, however, remains for future research.

## Conclusion

Our data revealed a complex and dynamic microbial community attached to the hindgut wall of the forest cockchafer. The composition of this community may be dependent on host’s life stage. L3 larvae showed a more close community to the adults than L2 larvae. In addition, the presence of particular bacterial niches attached to the larval hindgut (pockets) is reported. Regarding the surrounding hindgut wall, these niches harbored a differentiated bacterial community in which the families Micrococcaceae and Alcaligenaceae were dominant. These structures could be related to denitrification processes. Furthermore, the presence of poly-β-hydroxybutyrate (PHB) granules among pocket bacteria is demonstrated. Further research is needed to fully understand the function of the pockets, and especially to determine the role(s) of the cytoplasmatic inclusions.

## Author Contributions

PA-P performed DNA extraction, light microscopy, pyrosequencing data analysis, gas chromatography measurements, isolation, identification and metabolic testing of symbiotic bacteria and prepared the samples for Raman analysis. Also wrote the manuscript. EA-C spotted the pockets in the gut. Performed light microscopy, DNA extraction, fluorescence *in situ* hybridization, pyrosequencing data analysis and TEM data analysis. Also contributed in writing the manuscript. AN designed and performed qPCR experiments and analyzed the data. Also contributed in writing the manuscript. CE performed the Raman micro-spectroscopy analysis and analyzed the data. Also contributed in writing the manuscript. JR contributed in the design of TEM experiments and in the analysis of the data. MK contributed in the analysis of pyrosequencing data and calculated richness indexes and rarefaction curves. Also contributed in writing the manuscript. MW prepared samples for TEM, performed analysis and contributed in the analysis of the data. Spotted PHB inclusions in TEM images. UN contributed in the analysis of the Raman data. Also contributed in writing the manuscript. WB had the main idea of the project and supervised it. Also contributed in writing the manuscript.

## Conflict of Interest Statement

The authors declare that the research was conducted in the absence of any commercial or financial relationships that could be construed as a potential conflict of interest.

## Footnotes

1 www.researchandtesting.com/

2 http://greengenes.lbl.gov/

3 http://www.geneious.com

4 http://www.arb-silva.de
