# Supplementary material for: Bacterial Community and PHB-Accumulating Bacteria Associated with the Wall and Specialized Niches of the Hindgut of the Forest Cockchafer (Melolontha hippocastani)
Source: Front Microbiol. 2017 Feb 28;8:291. doi: 10.3389/fmicb.2017.00291 (PMC5329036; doi:10.3389/fmicb.2017.00291)
Supplement: Supplementary file 1 [file Data_Sheet_1.DOCX]

Supplementary Material

**Bacterial community and PHB-accumulating bacteria associated with the wall and specialized niches of the hindgut of the forest cockchafer (*Melolontha hippocastani*)**

**Pol Alonso-Pernas^*^, Erika Arias-Cordero, Alexey Novoselov, Christina Große, Jürgen Rybak, Martin Kaltenpoth, Martin Westermann, Ute Neugebauer, Wilhelm Boland^*^**

*** Correspondence:** Pol Alonso-Pernas: [palonso@ice.mpg.de](mailto:palonso@ice.mpg.de), Wilhelm Boland: [boland@ice.mpg.de](mailto:boland@ice.mpg.de)

## Supplementary Figures.


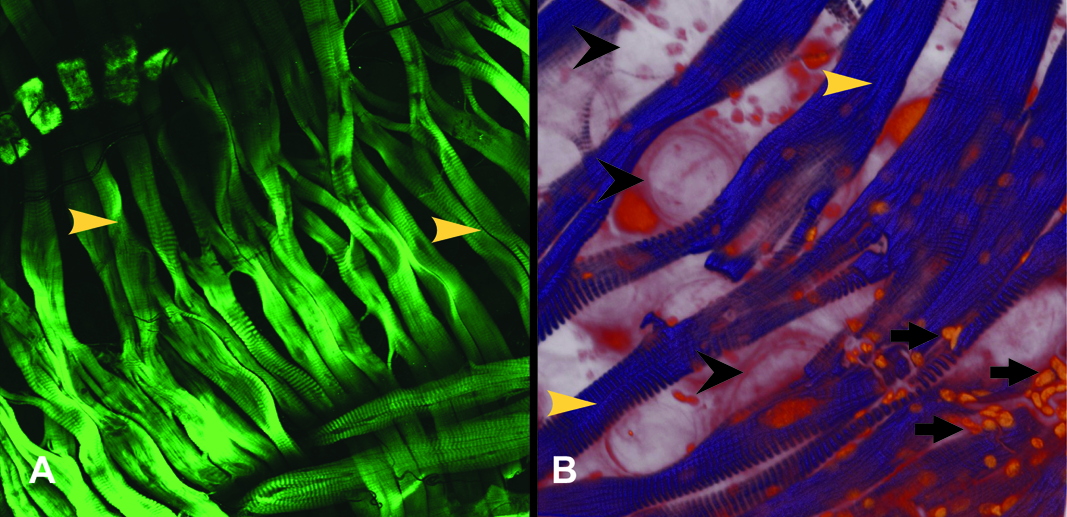


**Supplementary Figure 1**. Confocal images of the hindgut pocket tissue of a *Melolontha hippocastani* L2 larva. (A) Staining of the pocket tissue with Alexa Fluor 488 nm phalloidin stain (Phallotoxin, Invitrogen). (B) Double staining with Alexa Fluor 488 nm phalloidin stain and SYTOX Orange nucleic acid stain (Invitrogen), overlaid image. Yellow arrowheads point to muscle fibers that cover the pocket poles; black arrowheads indicate the position of the spheres at the distal point of the poles composing the pocket; black arrows point to the tracheoles that cover the pocket tissue.


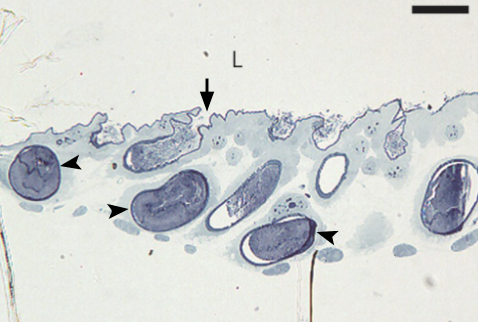


**Supplementary Figure 2**. *Melolontha hippocastani* L2 larva hindgut and pocket microscopic detail. Cross section stained with Richards’ solution. Black arrow point to the connection of one of the pocket poles to the hindgut lumen (L). Black arrowheads point to pocket poles. Scale bar 200 µm.


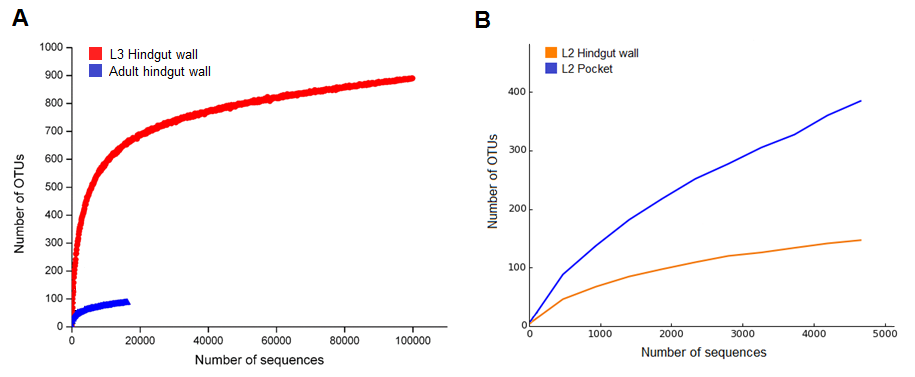


**Supplemetary Figure 3.** Rarefaction curves of the 454-pyro sequencing. (A) Comparison of L3 hindgut wall and adults hindgut wall. (B) Comparison of L2 hindgut wall and L2 pocket.

**
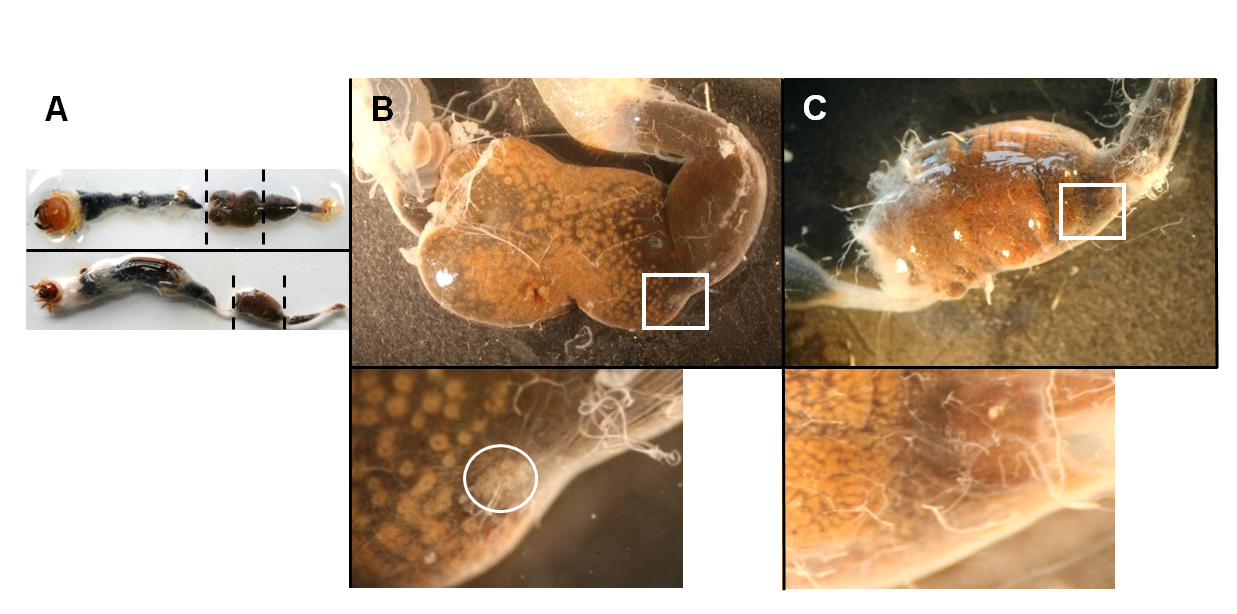
**

**Supplementary Figure 4.** Absence of pockets in *P. marginata* larvae compared to in *M. hippocastani*. The area within the white square is enlarged beneath. (A) Overview of a whole larval gut of *M. hippocastani* (upper image) and *P. marginata* (lower image). The hindgut chamber is between the dashed lines. (B) Close-up of *M. hippocastani* hindgut chamber. The pocket is inside the white circle in the enlarged image. (C) Close-up of *P. marginata* hindgut chamber. Note the absence of pocket in the enlarged image.

## Supplementary Tables.

**Supplementary Table 1**. Richness and diversity indices calculated at the OTU level from the pyrosequencing data of samples of pocket and hindgut wall of *Melolontha hippocastani*. Simpson expressed as 1-D, the bigger the number, the greater the diversity.

| **Sample** | **Total number of high quality reads** | **OTUs** | **Richness index** | **Diversity indexes** | |
| --- | --- | --- | --- | --- | --- |
|  |  |  | **Chao1** | **Shannon** | **Simpson** |
| Adults hindgut wall | 16 016 | 74 | 105.67 | 3.06 | 0.71 |
| L3 hindgut wall | 85 233 | 572 | 705.91 | 6.52 | 0.96 |
| L2 hindgut wall | 4 797 | 147 | 217.38 | 2.87 | 0.67 |
| L2 pocket | 4 726 | 889 | 1338.04 | 4.19 | 0.78 |

**Supplementary Table 2.** Genus-specific primers used.

| **Primer** | **Target** | **Sequence (5'-3')** | **Reference** |
| --- | --- | --- | --- |
| Achro F | *Achromobacter* spp. | GCTAATACCGCATACGCCCT | This study |
| Achro R | *Achromobacter* spp. | AGCCGTTACCCCACCAACTA | This study |
| Bos F | *Bosea* spp. | TAAGTTGGGAACTCTAGGGGG | This study |
| Bos R | *Bosea* spp. | TTTCGCTGCCCATTGTCACCG | This study |
| Brev F | *Brevundimonas* spp. | TTAGTTGGGAACTCTAATGG | This study |
| Brev R | *Brevundimonas* spp. | AGGATTAACCCTCTGTAGTTG | This study |
| Citro F | *Citrobacter* spp. | ACGGGTGAGTAATGTCTGGG | This study |
| Citro R | *Citrobacter* spp. | AGGTCCCCCTCTTTGGTCTT | This study |
| Pseudo F | *Pseudomonas* spp. | TTCGATTCAGCGGCGGACGG | This study |
| Pseudo R | *Pseudomonas* spp. | AGGTCCCCTGCTTTCTCCCGT | This study |

**Supplementary Table 3.** Abundance of “Low abundance families” expressed as a percentage of total sample sequences. N.D.: not detected.

| **Family** | **Pocket L2** | **Hindgut wall L2** | **Hindgut wall L3** | **Hindgut wall Adult** |
| --- | --- | --- | --- | --- |
| Procabacteriaceae | 0.0261 | N.D. | 0.1237 | N.D. |
| Veillonellaceae | N.D. | N.D. | 0.1396 | N.D. |
| Proteobacteria phylum unk. fam. | 0.1329 | N.D. | N.D. | N.D. |
| Gammaproteobacteria class unk. fam. | N.D. | N.D. | 0.1325 | N.D. |
| Oxalobacteraceae | 0.0057 | 0.0202 | 0.0721 | 0.0107 |
| Microbacteriaceae | 0.0403 | N.D. | 0.0545 | N.D. |
| Nocardioidaceae | N.D. | 0.0927 | N.D. | N.D. |
| Bacteroidaceae | 0.0591 | 0.0300 | N.D. | N.D. |
| Opitutaceae | N.D. | N.D. | 0.0708 | N.D. |
| Chitinophagaceae | 0.0641 | N.D. | N.D. | N.D. |
| Methylobacteriaceae | 0.0169 | 0.0395 | N.D. | N.D. |
| Peptococcaceae | N.D. | N.D. | 0.0514 | N.D. |
| Turicibacteraceae | 0.0438 | N.D. | N.D. | N.D. |
| Bradyrhizobiaceae | 0.0095 | 0.0202 | N.D. | 0.0027 |
| Phyllobacteriaceae | 0.0297 | N.D. | N.D. | N.D. |
| Propionibacteriaceae | 0.0019 | 0.0202 | N.D. | N.D. |
| Moraxellaceae | 0.0095 | 0.0102 | N.D. | N.D. |
| Patulibacteraceae | 0.0114 | N.D. | N.D. | N.D. |
| Catabacteriaceae | 0.0114 | N.D. | N.D. | N.D. |
| Rhodobacteraceae | 0.0114 | N.D. | N.D. | N.D. |
| Staphylococcaceae | N.D. | 0.0102 | N.D. | N.D. |
| Coriobacteriaceae | 0.0076 | N.D. | N.D. | N.D. |
| Bacteroidia class unk. fam. | N.D. | N.D. | 0.0005 | 0.0027 |

**Supplementary Table 4.** Raman bands assignment. Slashes (/) indicate different band positions with the same assignment. Hyphens (-) indicate interval. def.: deformation.

| **Observed band (cm^-1^)** | **Band assignment** | **References** |
| --- | --- | --- |
| 500 / 505 | S-S stretch | (Tuma 2005),(Maquelin et al. 2002) |
| 650 | C-C twist Tyr | (Tuma 2005),(Maquelin et al. 2002),(Neugebauer et al. 2010) |
| 837 / 841 | C-C stretch | (Majed and Gu 2010),(Ciobotǎ et al. 2010) |
| 854 / 859 | Ring vibration Tyr | (Tuma 2005),(Maquelin et al. 2002) |
| 1007 | Phenylalanine | (Tuma 2005),(Maquelin et al. 2002),(Neugebauer et al. 2010) |
| 1058 / 1063 / 1067 | C-O and C-C stretches | (Majed and Gu 2010),(Ciobotǎ et al. 2010),(Wu et al. 2011) |
| 1114 | C-C stretch | (Wu et al. 2011) |
| 1131 | C-O-H def., C-O and C-C stretches. | (Wu et al. 2011) |
| 1152 | C-N and C-C stretches | (Neugebauer et al. 2010), (Notingher et al. 2003) |
| 1240 - 1280 | C-H_2_ twist, amide III | (Wu et al. 2011),(Tuma 2005),(Maquelin et al. 2002),(Neugebauer et al. 2010),(Notingher et al. 2003) |
| 1440 / 1470 | C-H deformation | (Wu et al. 2011),(Tuma 2005),(Maquelin et al. 2002) |
| 1622 / 1626 | C=C stretch Tyr and Trp | (Maquelin et al. 2002),(Notingher et al. 2003) |
| 1650 - 1680 | amide I, C=C stretch | (Tuma 2005),(Maquelin et al. 2002),(Notingher et al. 2003), |
| 1729 / 1741 | C=O stretch | (Ciobotǎ et al. 2010),(Majed and Gu 2010),(Wu et al. 2011) |
| 2800 - 3000 | C-H_2_ and C-H_3_ sretches | (Majed and Gu 2010),(Ciobotǎ et al. 2010),(Wu et al. 2011),(Maquelin et al. 2002) |
